# Activation of Nuclear Factor Erythroid 2-Related Factor-2 by Oxylipin from *Mangifera indica* Leaves

**DOI:** 10.3390/antiox13091119

**Published:** 2024-09-16

**Authors:** Atif Ali Khan Khalil, Min-Seok Woo, Dong-Min Kang, Mi-Jeong Ahn, Jeong-Ah Kim, Heejung Yang, Jung-Hwan Kim

**Affiliations:** 1 Department of Pharmacology, Institute of Health Sciences, School of Medicine, Gyeongsang National University, Jinju 52727, Republic of Korea; atif.ali@gnu.ac.kr (A.A.K.K.); minseokwoo@gnu.ac.kr (M.-S.W.); 2 Research Institute of Pharmaceutical Sciences, College of Pharmacy, Gyeongsang National University, Jinju 52828, Republic of Korea; kdm7105@gnu.ac.kr (D.-M.K.); amj5812@gnu.ac.kr (M.-J.A.); 3 Research Institute of Pharmaceutical Sciences, College of Pharmacy, Kyungpook National University, Daegu 41566, Republic of Korea; jkim6923@knu.ac.kr; 4 College of Pharmacy, Kangwon National University, Chuncheon 24341, Republic of Korea; heejyang@kangwon.ac.kr

**Keywords:** NRF2, *Mangifera indica* L., fatty oxylipin, antioxidant, ubiquitination

## Abstract

*Mangifera indica* L., a member of the Anacardiaceae family, is widely cultivated across the globe. The leaves of *M. indica* are renowned for their medicinal properties, attributed to the abundance of bioactive compounds. This study investigated the effects of mango leaf extract on oxidative stress in HeLa cells. Notably, the n-hexane fraction (MLHx) significantly enhanced antioxidant response element (ARE)-luciferase activity at a concentration of 100 µg/mL, surpassing other fractions. MLHx also promoted the expression of HO-1 mRNA by increasing nuclear NRF2 levels. The molecular mechanism of MLHx involves increased phosphorylation of ERK1/2 and stabilization of NRF2. Bioactivity-guided isolation resulted in the identification of six oxylipins: 13(R)-hydroxy-octadeca-(9Z,11E,15Z)-trienoic acid (**C-1**), 9(R)-hydroxy-octadeca-(10E,12Z,15Z)-trienoic acid (**C-2**), 13(R)-hydroxy-(9Z,11E)-octadecadienoic acid (**C-3**), 9(R)-hydroxy-(10E,12Z)-octadecadienoic acid (**C-4**), 9-oxo-(10E,12E)-octadecadienoic acid (**C-5**), and 9-oxo-(10E,12Z)-octadecadienoic acid (**C-6**). These structures were elucidated using comprehensive spectroscopic techniques, including MS and 1H NMR. Additionally, compounds **C-7** (9-oxo-(10E,12Z,15Z)-octadecatrienoic acid) and **8** (13-oxo-(9E,11E)-octadecadienoic acid) were characterized by LC-MS/MS mass fragmentation. This study reports the isolation of compounds 1–6 from *M. indica* for the first time. When tested for their effect on NRF2 activity in HeLa cells, compounds **3**, **5**, and **6** showed strong stimulation of ARE-luciferase activity in a dose-dependent manner.

## 1. Introduction

Nuclear factor erythroid 2-related factor 2 (NRF2) is an essential transcription factor in the cellular defense mechanism against oxidative stress and inflammation [1]. It regulates the expression of several cytoprotective and antioxidant genes, thereby protecting cells from harmful assaults such as reactive oxygen species (ROS) [2]. Diseases characterized by oxidative stress and chronic inflammation, such as cancer, cardiovascular disease, and neurodegenerative disorders, have garnered significant interest as potential therapeutic targets, including those aimed at activating NRF2 [3,4].

Under normal conditions, NRF2 levels are maintained in the cytoplasm by its interaction with KEAP1 (kelch-like ECH-associated protein 1), which targets NRF2 for ubiquitination and subsequent degradation in the proteasome. This balance is disturbed when NRF2 is stabilized by oxidative stress, electrophilic agents, or other stressors. Once stabilized, NRF2 translocates into the nucleus, where it binds to antioxidant response elements (AREs) in the promoter regions of specific genes. This binding activates the expression of numerous cytoprotective proteins [5,6,7,8,9,10,11,12]. Notable examples of target genes regulated by NRF2 include heme oxygenase-1 (HO-1), NAD(P)H: quinone oxidoreductase-1 (NQO-1), and glutamate–cysteine ligase catalytic subunit (GCLC). NRF2 induces the expression of these genes by binding to antioxidant response elements (AREs) in their promoter regions. [13,14]. Extensive studies have been conducted on the functional manipulation of NRF2, both through activation and inhibition. As previously stated, KEAP1 acts as a key inhibitor of NRF2 by promoting its degradation through the ubiquitin–proteasome pathway [15]. However, it has been demonstrated that a number of kinases can increase NRF2 activity, including IQGAP1, AMP-activated protein kinase α (AMPKα), protein kinase C (PKC), AKT, and mitogen-activated protein kinases (MAPKs), including p38 and ERK1/2 [16,17,18,19,20,21].

Recent research has highlighted the potential of natural substances in modulating the NRF2 pathway. These compounds, derived from various plants and dietary sources, can enhance NRF2 activity either by directly interacting with KEAP1 or by influencing other upstream signaling pathways. [22].

*Mangifera indica* L., commonly known as the mango tree, is renowned for its extensive nutritional and therapeutic uses. The leaves of *M. indica* are rich in bioactive compounds, while the fruit is also widely consumed and recognized for its health benefits [23]. Consequently, extracts from mango leaves have been used in traditional medicine to treat various gastrointestinal, respiratory, and urinary disorders [24,25]. In this study, we aimed to isolate and identify bioactive compounds from the 80% ethanol extract of *M. indica* leaves and to investigate the effect of extracts and isolated compounds on NRF2 activation in HeLa cells.

## 2. Materials and Methods

### 2.1. Plant Materials

The *M. indica irwin* leaves were obtained from a greenhouse in Kumsan (Coordinates: 36.1208114″ N, 127.4656049″ E), Chungnam, South Korea, in December 2023. The voucher specimens (PGSC No. 701) were deposited in the herbarium of the College of Pharmacy, Gyeongsang National University.

### 2.2. Chemical Analysis

We acquired ^1^H NMR spectra using Bruker DRX-300 and 400 spectrometers (Karlsruhe, Germany). The Cambridge Isotope Laboratories, Inc. (Andover, MA, USA) provided NMR solvents. The chemical shifts were reported as δ (ppm) values.

LC-MS data were collected using a SCIEX X500R QTOF MS instrument with an electrospray ionization source operating in negative ion mode (ESI-negative) (Framingham, MA, USA). The ion source voltage was set at −4500 V. For both gas 1 and gas 2, the ion source gases were adjusted to a pressure of 50 psi. The curtain gas pressure was maintained at 30 psi, while the ion source temperature was set at 500 °C. The mass spectrometer functioned in IDA mode, covering a Time-of-Flight Mass Spectrometry (TOF MS) range of 100–1500 Daltons (Da) and an MS/MS range of 50–1500 Da. An initial DP of −80 V and a collision energy (CE) of −35 ± 15 V were established. Acquisition and analysis of data were conducted using SCIEX OS software, specifically version 3.0.0.3339.

Medium pressure liquid chromatography, which is also called MPLC, was performed using a 25 g sample of YMC GEL ODS-A (12 nm, S-150 μM) from YMC Co. Ltd. (Kyoto, Japan) with the Biotage Isolera One system (Charlotte, NC, USA). The MPLC was run at room temperature with a gradient of distilled water and methanol (100:0–0:100), and the UV detection was accomplished at 254 nm. The flow rate was 15 mL/min.

Preparative high-performance liquid chromatography (Prep-HPLC) was conducted with a Gilson Trilution System equipped with a YMC-Pack ODS-A column (250 × 20 mm I.D., S-5 µm, 12 nm) from YMC Co. Ltd. (Kyoto, Japan) and manufactured by Gilson, Inc. (Middleton, WI, USA). The prep-HPLC was run at room temperature with a gradient elution using solvent A (water) and solvent B (acetonitrile). The solvent system is as follows: 64% acetonitrile to 95% from 0 to 12 min. After that, a conditioning phase was utilized to bring the column back to its initial state for a period of five minutes. The UV detection was accomplished at a wavelength of 254 nm, and the flow rate was 4 mL/min.

Thin layer chromatography (TLC) was carried out using silica gel 60 F254 plates (Merck, Darmstadt, Germany). Compounds were visualized by exposing the TLC plates to ultraviolet (UV) light. TLC was performed with an elution of chloroform and methanol mixture (10:1), and the spots were visualized with UV 254 nm.

Optical rotations were measured with a JASCO P2000 polarimeter (Jasco, Tokyo, Japan). All chemicals utilized in the bioassay were of biochemical reagent grade.

### 2.3. Extraction and Isolation of Chemicals

The air-dried leaves of *M. indica* (2.8 Kg) were pulverized and subjected to extraction using 80% ethanol at ambient temperature. And then the extract was subjected to filtration. After filtration, a rotary evaporator was utilized in order to make the extract concentrated. The crude extract (727.8 g) obtained was mixed with water and separated into different fractions using organic solvents such as n-hexane, ethyl acetate (EtOAc), and n-butanol (n-BuOH) in sequence. This process successively yields a n-hexane Fr. (64 g), an EtOAc Fr. (111.2 g), a n-BuOH Fr. (183 g), and a water Fr. (311 g).

The hexane fraction was fractionated by reverse phase medium pressure liquid chromatography (RP-MPLC) eluted with a gradient of water and methanol to yield five subfractions (fr.1–fr.5). Further chromatography was performed on the oxylipin-rich subfraction fr.2 using preparative high-performance liquid chromatography (prep-HPLC) with gradient elution. The solvent system consisted of water (A) and acetonitrile (B), as follows: 64% acetonitrile to 95% from 0 to 12 min. The solvent B returned to its initial condition at 17 min. The UV detection was accomplished at a wavelength of 254 nm, and the flow rate was 4 mL/min. This prep-HPLC afforded a total of eight subfractions (fr.2.1–fr.2.8). A subfraction fr.2.4 was subjected to preparatory TLC (aluminium plat; 20 × 20 cm) in a glass TLC chamber. Which yielded compound **C-1** (53 mg) and **C-2** (5 mg) by repeated preparatory TLC with an elution of chloroform and methanol mixture (10:1). Compounds **C-3** (17.5 mg) and **C-4** (27 mg) were isolated from fr. 2.6, by preparatory TLC under the same conditions.

Compounds **C-5** (3.5 mg) and **C-6** (2.4 mg) were isolated with the help of an Agilent 1260 series liquid chromatography (LC) system from Agilent Technologies (Palo Alto, CA, USA). This system was equipped with an autosampler, a binary pump, a column oven, and a degasser. An aliquot (25 μL) of sample solution of fraction 2.7 (KODE rich fraction) was directly injected on a Hypersil C18 column of thermos scientific (4.6 × 250 mm, 5 μm) equipped with a compatible guard column. The components were resolved by gradient elution using an acetonitrile and water solvent system as follows: 64% acetonitrile to 95% from 0 to 12 min. After that, a conditioning phase was utilized to bring the column back to its initial state for a period of five minutes. The flow rate was 1 mL/min, and the column temperature was 30 °C. This preparative LC afforded compound **C-5** (3.5 mg, tR = 9.68 min) and **C-6** (2.5 mg, tR = 10.19 min).

13(R)-hydroxy-octadeca-(9Z,11E,15Z)-trienoic acid (**C-1**): Yellow oil; [α]$\begin{array}{c} 20\\ D \end{array}$ -4.6 (CH_3_OH, c = 0.03). HREI-MS *m*/*z* 293.2118 [M-H]^-^ (calcd for C_18_H_30_O_3_, 294.2196). ^1^H NMR (400 MHz, MeOD) *δ* 6.52 (1H, dd, *J* = 15.2, 11.0 Hz, H-11), 5.99 (1H, t, *J* = 11.0 Hz, H-10), 5.66 (1H, dd, *J* = 15.2, 6.5 Hz, H-12), 5.50 (1H, dt, *J* = 10.9, 7.4 Hz, H-16), 5.43 (1H, dt, *J* = 10.9, 7.0 Hz, H-9), 5.37 (1H, dt, *J* = 10.9, 7.4 Hz, H-15), 4.13 (1H, q, *J* = 6.5 Hz, H-13), 2.32 (2H, t, *J* = 7.4 Hz, H-2), 2.28 (2H, t, *J* = 7.4 Hz, H-14), 2.21 (2H, q, *J* = 7.0 Hz, H-8), 2.08 (2H, quint, *J* = 7.4 Hz, H-17), 1.62 (2H, m, H-3), 1.42 (2H, m, H-7), 1.35 (6H, m, H-4, 5, 6), 0.98 (3H, t, *J* = 7.4 Hz, H-18) [26,27].

9(R)-hydroxy-octadeca-(10E,12Z,15Z)-trienoic acid (**C-2**): colorless oil; [α]$\begin{array}{c} 20\\ D \end{array}$ -12.5 (CH_3_OH, c = 0.03). HREI-MS *m*/*z* 293.2117 [M-H]^-^ (calcd for C_18_H_30_O_3_, 294.2196). ^1^H NMR (400 MHz, MeOD) *δ* 6.55 (1H, dd, *J* = 15.0, 11.0 Hz, H-11), 6.00 (1H, t, *J* = 11.0 Hz, H-12), 5.67 (1H, dd, *J* = 15.0, 7.0 Hz, H-10), 5.43 (1H, dt, *J* = 11.0, 6.7 Hz, H-16), 5.38 (1H, dt, *J* = 11.0, 6.7 Hz, H-13), 5.33 (1H, dt, *J* = 11.0, 6.7 Hz, H-15), 4.10 (1H, q, *J* = 6.7 Hz, H-9), 2.96 (2H, t, *J* = 7.4 Hz, H-14), 2.32 (2H, m, H-2), 2.11 (2H, quint, *J* = 7.4 Hz, H-17), 1.62 (2H, m, H-3), 1.54 (2H, m, H-8), 1.45 (2H, m, H-6), 1.35 (6H, m, H-4, 5, 7), 1.00 (3H, t, *J* = 7.4 Hz, H-18) [26,28].

13(R)-hydroxy-(9Z,11E)-octadecadienoic acid (**C-3**): colorless oil; [α]$\begin{array}{c} 20\\ D \end{array}$ -7.5 (CH_3_OH, c = 0.03). HREI-MS *m*/*z* 295.2278 [M-H]^-^ (calcd for C_18_H_32_O_3_, 296.2353). ^1^H NMR (400 MHz, MeOD) *δ* 6.51 (1H, dd, *J* = 15.0, 11.0 Hz, H-11), 5.99 (1H, t, *J* = 11.0 Hz, H-10), 5.63 (1H, dd, *J* = 15.0, 6.6 Hz, H-12), 5.42 (1H, td, *J* = 11.0, 7.6 Hz, H-9), 4.09 (1H, q, *J* = 6.6 Hz, H-13), 2.30 (2H, t, *J* = 6.0 Hz, H-2), 2.21 (2H, q, *J* = 7.6 Hz, H-8), 1.61 (2H, m, H-3), 1.52 (2H, m, H-14), 1.42 (2H, m, H-7), 1.35 (12H, m, H-4, 5, 6, 15, 16, 17), 0.93 (3H, t, *J* = 7.0 Hz, H-18) [29,30].

9(R)-hydroxy-(10E,12Z)-octadecadienoic acid (**C-4**): colorless oil; [α]$\begin{array}{c} 20\\ D \end{array}$ -7.2 (CH_3_OH, c = 0.03). HREI-MS *m*/*z* 295.2277 [M-H]^-^ (calcd for C_18_H_32_O_3_, 296.2353). ^1^H NMR (400 MHz, MeOD) *δ* 6.51 (1H, dd, *J* = 15.0, 11.0 Hz, H-11), 5.99 (1H, t, *J* = 11.0 Hz, H-12), 5.63 (1H, dd, *J* = 15.0, 6.8 Hz, H-10), 5.43 (1H, dt, *J* = 11.0, 7.6 Hz, H-13), 4.09 (1H, q, *J* = 6.8 Hz, H-9), 2.30 (2H, t, *J* = 7.0 Hz, H-2), 2.21 (2H, q, *J* = 7.0 Hz, H-14), 1.62 (2H, m, H-3), 1.52 (2H, m, H-8), 1.42 (2H, m, H-7), 1.32 (12H, m, H-4, 5, 6, 15, 16, 17), 0.93 (3H, t, *J* = 6.9 Hz, H-18) [29,31].

9-Oxo-(10E,12Z)-octadecadienoic acid (**C-5**): pale yellow oil; HREI-MS *m*/*z* 293.2120 [M-H]^-^ (calcd for C_18_H_30_O_3_, 294.2196). ^1^H NMR (300 MHz, MeOD) *δ* 7.60 (1H, dd, *J* = 15.1, 11.5 Hz, H-11), 6.23 (1H, d, *J* = 15.1 Hz, H-10), 6.21 (1H, t, *J* = 11.5 Hz, H-12), 5.99 (1H, dt, *J* = 11.5, 7.5 Hz, H-13), 2.63 (2H, t, *J* = 7.5 Hz, H-8), 2.35 (2H, m, H-2), 2.26 (2H, m, H-14), 1.62 (2H, m, H-3), 1.48 (2H, m, H-7), 1.36 (12H, m, H-4, 5, 6, 15, 16, 17), 0.94 (3H, t, *J* = 7.4 Hz, H-18) [32,33].

9-Oxo-(10E,12E)-octadecadienoic acid (**C-6**): pale yellow oil; HREI-MS *m*/*z* 293.2122 [M-H]^-^ (calcd for C_18_H_30_O_3_, 294.2196). ^1^H NMR (300 MHz, MeOD) *δ* 7.25 (1H, dm, *J* = 15.5 Hz, H-11), 6.30 (1H, m, H-12, 13), 6.14 (1H, d, *J* = 15.5, H-10), 2.61 (2H, t, *J* = 7.5 Hz, H-8), 2.30 (2H, m, H-2), 2.21 (2H, m, H-14), 1.61 (2H, m, H-3), 1.48 (2H, m, H-7), 1.33 (12H, m, H-4, 5, 6, 15, 16, 17), 0.93 (3H, t, *J* = 7.0 Hz, H-18) [32,33].

9-oxo-(10E,12Z,15Z)-octadecatrienoic acid (**C-7**): The structure of compound **7** was confirmed with previously reported mass data. The 9-oxo-10,12,15-octadecatrienoic acid peak was detected as an unfragmented deprotonation ion (C_18_H_27_O_3_−, [M-H]^-^, Supplementary data) by electrospray negative ionization mass spectrometry. The fragment ions detected (*m*/*z* = 185, 125, and 121) matched with the previously reported data [34].

13-oxo-(9E,11E)-octadecadienoic acid (**C-8**): The compound **8** MS/MS peaks were in good agreement with that of previously reported mass data. The 13-oxo-9,11-octadecadienoic acid peak was detected as an unfragmented deprotonation ion (C_18_H_29_O_3_−, [M-H]^-^, Supplementary data) by electrospray negative ionization mass spectrometry. The fragment ions detected (*m*/*z* = 249, 195, and 113) matched with the previously reported data [32,33].

### 2.4. Cell Culture and Reagents

HeLa cells used in the current study were obtained from the American Type Culture Collection (ATCC) and cultured in DMEM medium supplemented with 10% fetal bovine serum and an antibiotic–antimycotic solution. The solution included penicillin (100 units/mL), streptomycin (100 µg/mL), and amphotericin B (0.25 µg/mL). Cultivation occurred in a humidified incubator set at 37 °C with 5% CO_2_ and 95% air. The cellular confluence rate remained consistently between 60% and 70% throughout all experiments. Antibodies used for western blotting were anti-NRF2 (cell signaling Cat#5412, RRID: AB_564548), anti-HO-1 (Santa Cruz Cat#48565, RRID: AB_7859), anti-Lamin A/C (Santa Cruz Biotechnology), anti-GFP (Santa Cruz Biotechnology), and anti-GAPDH (Abcam Cat#12345, RRID: AB_12458).

### 2.5. Cell Toxicity Assay

The MTT assay was performed to assess the cytotoxic effects of the tested materials on HeLa cells, following the previously reported method [35]. In detail, cells were treated with various concentrations (0–200 μM) for 24 h, with DMSO concentrations kept below 0.2%. After treatment, 20 µL of MTT solution (5 mg/mL) was added to each well and incubated for two hours. Formazan crystals were dissolved with 150 µL of DMSO, and absorbance at 570 nm was measured using a Tecan plate reader (Tecan instrument, Morgan Hill, CA, USA). All experiments were performed in triplicate and repeated at least twice for accuracy.

### 2.6. ARE Luciferase Assay

The effect of each isolated compound on ARE luciferase activity was evaluated in HeLa cells using the Dual-Luciferase Reporter Assay (Promega), according to the manufacturer’s instructions. Briefly, cells were seeded in 48-well plates and treated with various concentrations (0–200 μM) of the isolated compounds for 6 h, following overnight co-transfection with the pGL4.21 3 × ARE plasmid (100 ng/well) or the pGL4.10-2 kb-NRF2 promoter plasmid, along with the pRL-Renilla luciferase control vector (20 ng/well). After treatment, cells were lysed with 100 μL of 1X passive lysis solution (Promega, WI, USA) at room temperature. ARE luciferase activity was measured using 10 μL of the lysate and normalized to Renilla luciferase activity.

### 2.7. Western Blotting

Cytosolic and nuclear lysates from HeLa cells were prepared using M-PER buffer. Protein concentrations were determined with BCA reagent (Thermo Scientific, Waltham, MA, USA) at 570 nm. Proteins (30 µg) were separated on a 4–20% gradient SDS-PAGE and transferred to a nitrocellulose membrane using the Trans-Blot Turbo system (Bio-Rad, CA, USA). The membrane was blocked with 5% non-fat dry milk in PBS-T (0.1% Tween-20) for 1 h, incubated with primary antibodies overnight at 4 °C, and then with horseradish peroxidase-conjugated secondary antibodies for 1 h. Detection was performed using Bio-Rad ECL substrate on the ChemiDoc System.

### 2.8. Quantitative Real-Time PCR Analysis

Following the directions provided by the manufacturer, total RNA was extracted with the help of the TRIzol reagent (Invitrogen, Carlsbad, CA, USA). Subsequently, the qScript cDNA Synthesis Kit (QuantaBio, Beverly, MA, USA) was used to synthesize cDNA, which was then utilized for qPCR. The qPCR reaction utilized PerfeCTa SYBR Green FastMix (QuantaBio, Beverly, MA, USA) under the following conditions: an initial step was at 95 °C for 30 s, followed by 45 cycles, which were at 95 °C for 5 s and 60 °C for 10 s. The reaction was then cooled at 4 °C for 10 s using the QuantStudioTM 5 instrument (Thermo Scientific, MA, USA). The following primer sets were used for the qPCR analysis: NRF2-F, 5′-TCT TGC CTC CAA AGT ATG TCA A-3′ and NRF2-R, 5′-ACA CGG TCC ACA GCT CAT C-3′; NQO-1-F, 5′-TCC TTT CTT CTT CAA AGC CG-3′; and NQO-R, 5′-GGA CTG CAC CAG AGC CAT-3′; HO-1-F, 5′-GAG TGT AAG GAC CCA TCG GA-3′ and HO-R, 5′-GCC AGC AAC AAA GTG CAA G-3′; GAPDH-F, 5′-AAG GTG AAG GTC GGA GTC AA-3′ and GAPDH-R, 5′-AAT GAA GGG GTC ATT GAT GG-3′. Following amplification, reactions were cooled at 4 °C for 10 s. All reactions were conducted in triplicate to ensure accuracy and reproducibility. Accession numbers of genes are as follows: HO-1: NM_002133, NRF2: NM_006164, NQO1: NM_000903, GAPDH: NM_001256799.

### 2.9. Protein Stability Assay

HeLa cells were transfected with 3 μg of pcDNA4-His-Ubi and 5 μg of pEGFP-Nrf2 plasmids using a linear polyethylenimine (PEI, Sigma-Aldrich, MO, USA) reagent for 24 h. Following transfection, the cells were treated with 100 μg/mL of MLHx for 6 h. After treatment, cells were harvested using RIPA lysis buffer to obtain whole-cell protein extracts. A total of 400 μg of whole-cell extract was incubated with 100 μL of Ni-NTA beads at 4 °C for 1 h on a rotary shaker to allow for binding. The bead-protein complexes were then washed three times with RIPA buffer for 5 min at 4 °C on a rotary shaker. After washing, the bead-protein complexes were centrifuged at 1000× *g* for 1 min. The beads were resuspended in 65 μL of 2× Laemmli sample buffer and boiled for 5 min. The samples were then analyzed by Western blotting to assess protein stability.

### 2.10. Statistical Analysis

The values obtained from the experiment were expressed as mean ± SD. In order to conduct the statistical analysis, a two-tailed Student’s *t*-test was applied for unpaired data. Furthermore, a *p* < 0.05 was determined to be statistically significant.

## 3. Results

### 3.1. MLHx Increases the NRF2 Activity through the ARE System in HeLa Cells

**Figure 1:**
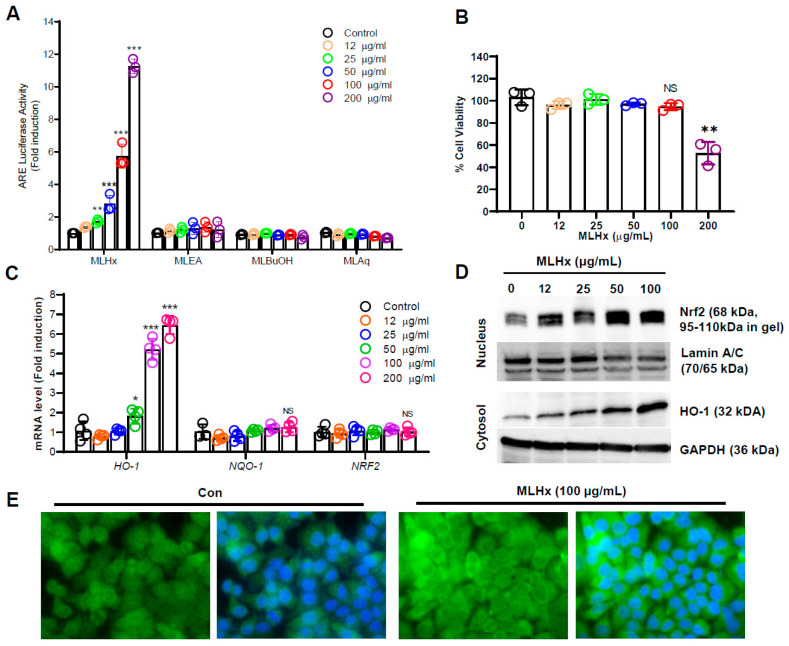
Effects of MLHx on NRF2 activity and expression in HeLa cells. (**A**) ARE-luciferase activity was measured in HeLa cells after 6 h of treatment with various doses of MLHx. (**B**) Cytotoxic effects were assessed using an MTT assay following 24 h treatment with different concentrations of MLHx. (**C**) qPCR analysis of mRNA levels for HO-1, NQO-1, and NRF2 after 6 h exposure to various MLHx doses. (**D**) Western blot analysis was performed after treating cells with different doses of MLHx for 24 h. * *p* < 0.05; ** *p* < 0.001; *** *p* < 0.0001. (**E**) Immunocytochemistry analysis of NRF2 expression after treatment with 100 µg/mL MLHx. Scale bar, 20 µm.

To evaluate NRF2 activity in response to mango leaf fractions, an ARE-luciferase assay was performed in HeLa cells. The results showed that the n-hexane fraction of mango leaves (MLHx) caused a significant and dose-dependent increase in ARE-luciferase activity compared to other mango leaf fractions (Figure 1A). The MTT assay demonstrated that MLHx had a growth-inhibitory effect at a concentration of 200 μg/mL (Figure 1B). However, MLHx did not exhibit any significant cytotoxic effects at a concentration of 100 μg/mL. Additionally, qPCR was conducted to verify the induction of the NRF2 gene and its target genes, such as HO-1 and NQO-1. The results showed that only HO-1 mRNA levels increased with MLHx treatment (Figure 1C). Furthermore, treatment with 100 μg/mL MLHx significantly increased nuclear NRF2 protein levels and cytosolic HO-1 protein levels in a dose-dependent manner (Figure 1D). The effect of MLHx on NRF2 was further confirmed by immunocytochemistry (Figure 1E).

### 3.2. MLHx Enhances NRF2 Activity in HeLa Cells through ERK1/2 Signaling

**Figure 2:**
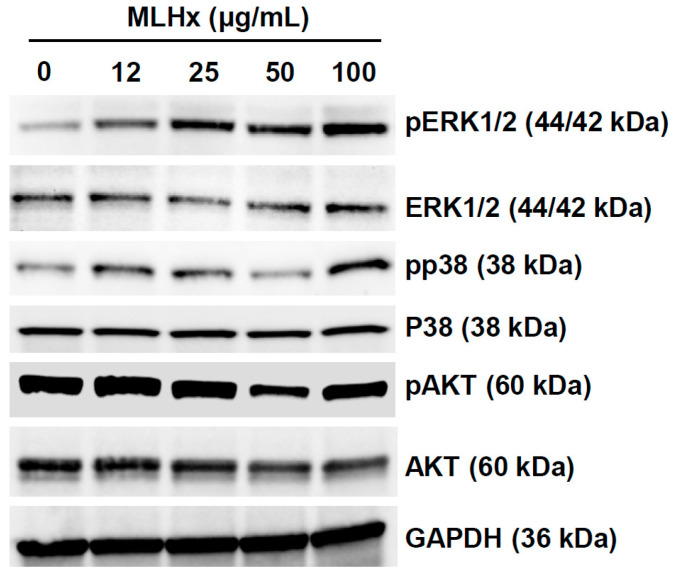
Activation of signaling pathways by MLHx in HeLa cells. Western blot analysis showing activation of AKT, p38, and ERK pathways in response to MLHx treatment for 3 h.

Given that mitogen-activated protein kinases (MAPKs) and AKT can stimulate NRF2, we investigated the impact of MLHx on MAPK activation. In a dose-dependent manner, MLHx significantly increased the phosphorylation of ERK1/2 within 3 h, as shown in Figure 2. However, MLHx did not increase AKT activation. Therefore, MLHx potentially stimulates NRF2 signaling activation via the ERK1/2 pathway.

### 3.3. MLHx Potentiates the NRF2 Stability by Inhibiting Ubiquitin-Mediated Degradation

**Figure 3:**
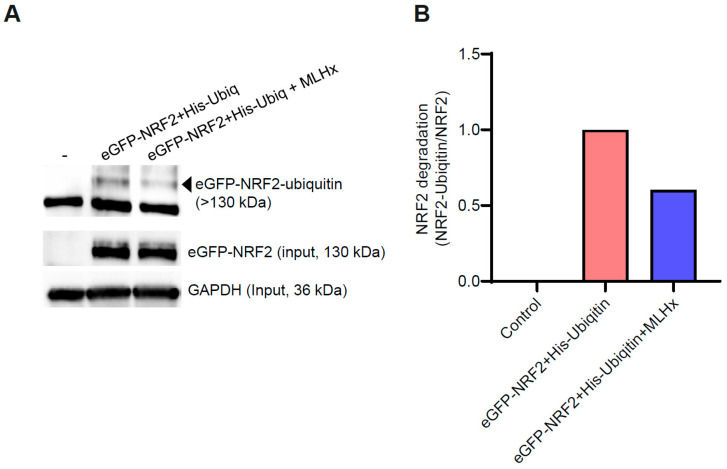
MLHx enhances NRF2 stability by inhibiting ubiquitin-mediated proteolysis in HeLa cells. (**A**) Cells were treated with 100 µg/mL of MLHx for 6 h after co-transfection with pEGFP-NRF2 and pcDNA3.1-His ubiquitin plasmids for 24 h. His-ubiquitinated proteins were purified using Ni-NTA agarose beads, and ubiquitinated eGFP-NRF2 was detected via Western blot analysis. (**B**) The relative fold change of ubiquitinated eGFP-NRF2 was quantified using densitometry. Experiments were performed in duplicate.

To investigate the effect of MLHx on NRF2 stability, which is typically regulated through ubiquitin-mediated proteasomal degradation, we used the EGFP-NRF2-Ubiquitin system. The cells were treated with 100 μg/mL of MLHx for 6 h while expressing EGFP-NRF2 and His-Ubiquitin. Upon Ni-NTA purification, we noted a significant reduction in the levels of ubiquitinated EGFP-NRF2 following MLHx treatment (Figure 3). This suggests that MLHx stabilizes NRF2 by inhibiting its ubiquitination.

### 3.4. Chemical Characterization

**Figure 4:**
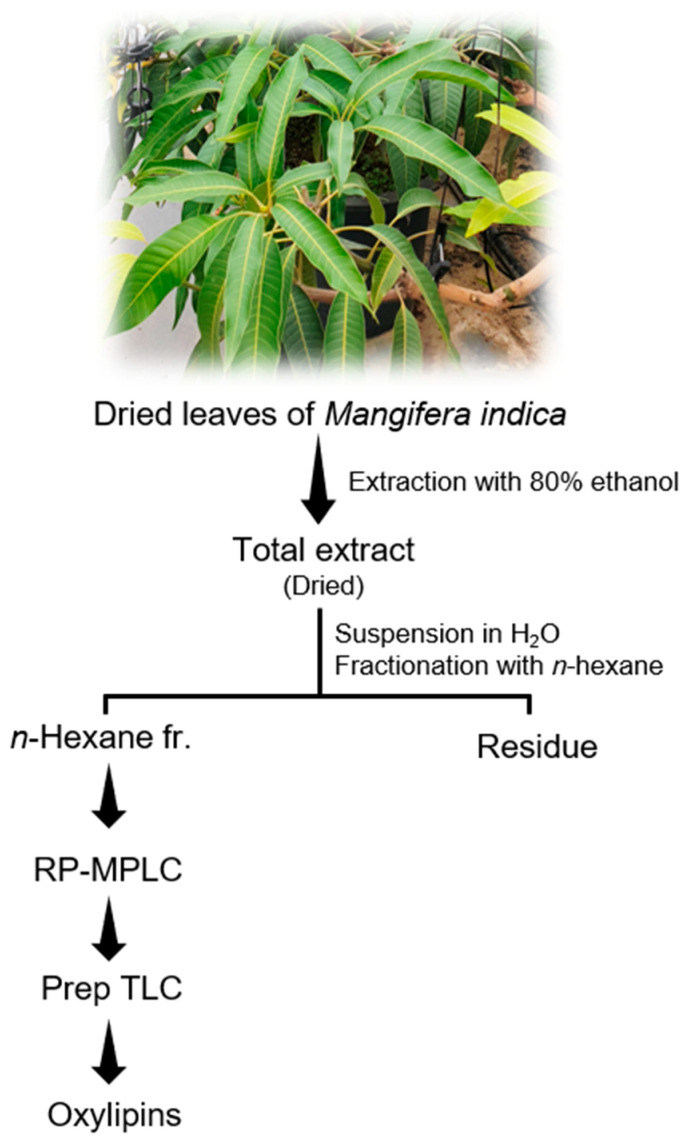
Process of oxylipin isolation from *Mangifera indica*.

**Figure 5:**
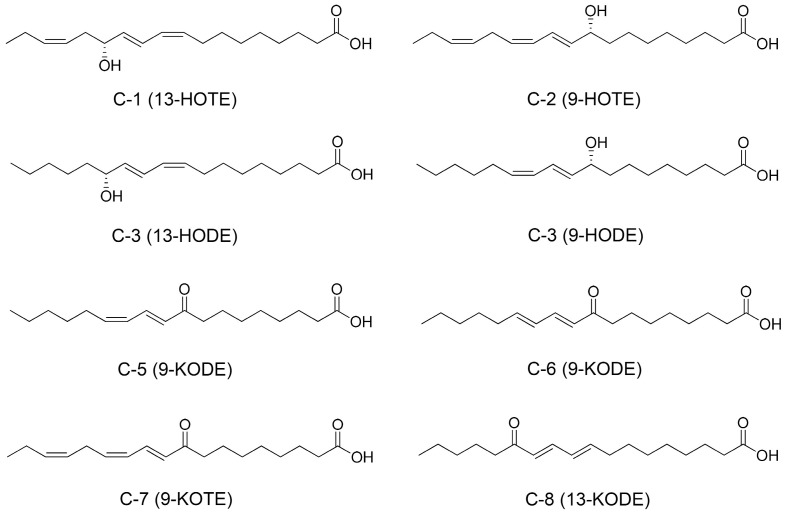
Chemical Structures of Isolated oxylipins.

A simple method for isolating and purifying oxylipin (hydroxyl- or oxo-fatty acid) was developed (Figure 4), resulting in the identification of six oxylipins (1–6) (Figure 5). The structures of these compounds were established using detailed spectroscopic data. The oxylipins were identified as 13(R)-hydroxy-octadeca-(9Z,11E,15Z)-trienoic acid (**C-1**), 9(R)-hydroxy-octadeca-(10E,12Z,15Z)-trienoic acid (**C-2**), 13(R)-hydroxy-(9Z,11E)-octadecadienoic acid (**C-3**), 9(R)-hydroxy-(10E,12Z)-octadecadienoic acid (**C-4**), 9-oxo-(10E,12E)-octadecadienoic acid (**C-5**), and 9-oxo-(10E,12Z)-octadecadienoic acid (**C-6**), as determined by spectroscopic analysis and comparison with previous studies (Supplementary data). In addition, the absolute configuration of compounds **C-1**, **C-2**, **C-3,** and **C-4** was determined. The specific rotational values of **C-1**, **C-2**, **C-3** and **C-4** were −4.6, −12.5, −7.5, and −7.2, respectively. The negative optical rotation values demonstrate that **C-1** and **C-3**, **C-2** and **C-4** at carbon 13 and 9 have the *R*-configuration, respectively [27,36,37]. Furthermore, compounds **C-7** and **C-8** were identified based on their mass fragmentation patterns and compared with previously reported data [32,33,34].

### 3.5. Isolated Oxylipins Increases the NRF2 Activity through the ARE System in HeLa Cells

**Figure 6:**
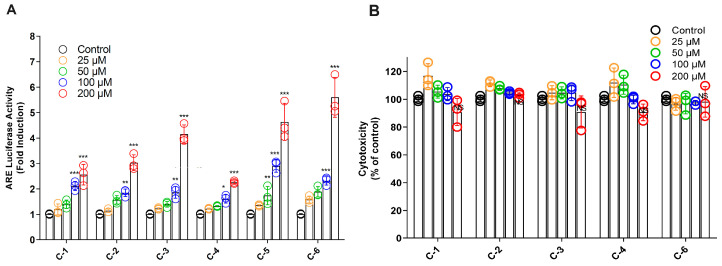
Biological activity of compounds 1–6 in HeLa cells. (**A**) ARE-luciferase activity was measured after 6 h treatment with indicated concentrations of compounds 1–6. (**B**) Cytotoxicity of compounds was evaluated using an MTT assay after 24 h treatment. * *p* < 0.05; ** *p* < 0.001; *** *p* < 0.0001.

Each isolated oxylipin was screened for ARE activation in HeLa cells using an ARE-luciferase reporter gene assay at doses ranging from 0 to 200 µM. Among the compounds, C-5 and C-6 exhibited potent activity, significantly stimulating luciferase activity in a dose-dependent manner. Compound C-3 also demonstrated substantial luciferase stimulation, ranking second compared to C-1, C-2, and C-4 (Figure 6A). Additionally, an MTT assay was achieved with different doses of the compounds (0–200 µM) for 24 h in HeLa cells. The results showed no significant cytotoxicity at the maximum dose of 200 µM (Figure 6B). However, compound C-5 could not be tested for cytotoxicity due to limited availability. These findings suggest that mango leaf fractions, particularly MLHx and its isolated oxylipins, enhance NRF2 activity, which could be beneficial for modulating oxidative stress.

## 4. Discussion

Due to the significant involvement of NRF2 in numerous diseases alongside oxidative stress, several studies have been conducted to explore the utilization of naturally occurring compounds as NRF2 activators. Alternatively, it could be advantageous to identify compounds that hinder the process of KEAP1-mediated NRF2 breakdown pathway [15].

Prioritizing the regulation of NRF2 is crucial in preventing the transformation of normal cells into abnormal ones, as numerous malignant cells have already acquired NRF2 activity as a means to evade excessive oxidative damage. Therefore, acquiring NRF2 activators could be advantageous as a strategy for chemotherapy prevention against many pathophysiological manifestations, such as cancer.

The purpose of this study is to report the molecular mechanisms underlying MLHx effects on NRF2 activation. In our study, MLHx treatment increased the nuclear expression of NRF2, resulting in activation of downstream target genes such as HO-1 in HeLA cells through utilization of ARE motifs. In addition, the ARE-luciferase activity of compounds isolated from *M. indica* is also investigated in this study. However, it is important to explore the NRF2 activity of crude extracts for different uses.

Not only is the signal transduction to NRF2 the most critical factor in maintaining the activity of NRF2, but the stability of NRF2 is also the most significant factor. Similarly, proteasomal degradation by ubiquitination is a representative example of the degradation system of NRF2, as reported previously [38,39]. Therefore, it is possible that MLHx could decrease the degradation of NRF2 by decreasing the degradation attributable to proteasomes.

Mechanistically, MAPKs, including p38 and ERK1/2, are probable mediators involved in MLHx-induced NRF2 activation [38]. Since MLHX only increased the phosphorylation of p38 and ERK1/2 without affecting AKT, suggesting that MLHX induced activation of NRF2 via modulation of MAPKs. In HeLa cells, MLHx reduced basal phosphorylation of AKT, suggesting that MLHx suppresses cell proliferation via this AKT modulation. Further, qPCR analysis revealed no effect of MLHx on transcript levels of NRF2, but it did affect MAPK activation, suggesting its involvement in the signaling pathway.

Further, activity-guided leads to the isolation of 6 oxylipins. The structures of these compounds were characterized through detailed spectroscopic data and comparison with previously reported literature [26,27,28,29,30,31,32,33,34]. The optical rotation of optically active compounds C-1, 2, 3, and 4 was measured using a polarimeter. The observed values verify our results in comparison to previously reported literature values [27,36,37]. Furthermore, we explored the ARE-luciferase activity of these isolated compounds from *M. indica*. Although ARE activity of compounds C-3, C-4, and C-5 has been previously reported [40,41]. The current study reveals that Oxo-ODE (C-5 and C-6) groups displayed better activity compared to HOTE (C-1 and C-2) and HODE (C-3 and C-4) groups, consistent with previously reported studies. Oxo-ODEs induce ARE activity 5–6-fold compared to HOTE and HODEs at 200 µM. This suggests that the addition of different functional groups to the oxylipin skeleton significantly affects ARE fold induction. Specifically, Oxo-ODEs, characterized by a ketone group at position nine, exhibited potent ARE activity compared to HOTEs and HODEs, which possess hydroxy groups at positions 9 and 13. The presence of these functional groups influences the overall activity of the compounds.

Oxylipins, including oxo-ODEs, HOTE, and HODE, are known to play critical roles in modulating various biological processes through their interaction with cellular signaling pathways. Oxo-ODEs, for instance, are recognized for their ability to activate the NRF2 pathway more effectively than HOTE and HODE, highlighting their potential as potent modulators of oxidative stress and inflammation [40]. The differences in functional groups, such as the ketone group in Oxo-ODEs versus the hydroxy groups in HOTEs and HODEs, contribute to the variations in their biological activity and effectiveness in NRF2 pathway modulation. In summary, our study concludes that the hexane fraction of *M. indica* leaves and isolated oxylipins, particularly those with oxylipin profiles, hold promise for chemoprevention or chemotherapeutic purposes. The ability of these compounds to modulate NRF2 activity and their distinct effects based on functional group composition underscore their potential utility in therapeutic applications targeting oxidative stress and related diseases.

## 5. Conclusions

Taken together, this study successfully isolated six oxylipins from the leaves of *M. indica*, marking the first successful extraction of these specific compounds from this plant. Notably, compounds **C-6**, **C-5**, and **C-3** demonstrated substantial enhancement of NRF2 activity, highlighting their potential as effective agents in modulating oxidative stress. The hexane fraction and the identified oxylipins derived from *M. indica* leaves show promise for therapeutic applications, particularly in protecting against oxidative stress through NRF2 pathway activation. These findings underscore the potential of *M. indica* as a source of bioactive compounds for developing treatments aimed at combating oxidative stress and related conditions.

## Data Availability

Data is contained within the article or Supplementary Material.

## Supplementary Materials

The following supporting information can be downloaded at: https://www.mdpi.com/article/10.3390/antiox13091119/s1.

## References

[1] Kim J., Cha Y.-N., Surh Y.-J. (2010). A protective role of nuclear factor-erythroid 2-related factor-2 (Nrf2) in inflammatory disorders. Mutat. Res./Fundam. Mol. Mech. Mutagen..

[2] He L., He T., Farrar S., Ji L., Liu T., Ma X. (2017). Antioxidants maintain cellular redox homeostasis by elimination of reactive oxygen species. Cell. Physiol. Biochem..

[3] Taha R., Blaise G. (2014). Nrf2 activation as a future target of therapy for chronic diseases. Funct. Foods Health Dis..

[4] Gallorini M., Carradori S., Panieri E., Sova M., Saso L. (2024). Modulation of NRF2: Biological dualism in cancer, targets and possible therapeutic applications. Antioxid. Redox Signal..

[5] Baird L., Yamamoto M. (2020). The Keap1-Nrf2 pathway: From mechanism to medical applications. Oxidative Stress.

[6] Kim J.-H., Xu E.Y., Sacks D.B., Lee J., Shu L., Xia B., Kong A.-N.T. (2013). Identification and functional studies of a new Nrf2 partner IQGAP1: A critical role in the stability and transactivation of Nrf2. Antioxid. Redox Signal..

[7] Chun K.-S., Kundu J., Kundu J.K., Surh Y.-J. (2014). Targeting Nrf2-Keap1 signaling for chemoprevention of skin carcinogenesis with bioactive phytochemicals. Toxicol. Lett..

[8] Park J.S., Kang D.H., Bae S.H. (2015). Fenofibrate activates Nrf2 through p62-dependent Keap1 degradation. Biochem. Biophys. Res. Commun..

[9] Kwak M.-K., Wakabayashi N., Kensler T.W. (2004). Chemoprevention through the Keap1–Nrf2 signaling pathway by phase 2 enzyme inducers. Mutat. Res./Fundam. Mol. Mech. Mutagen..

[10] Lee J.-M., Johnson J.A. (2004). An important role of Nrf2-ARE pathway in the cellular defense mechanism. BMB Rep..

[11] Nguyen T., Sherratt P.J., Pickett C.B. (2003). Regulatory mechanisms controlling gene expression mediated by the antioxidant response element. Annu. Rev. Pharmacol. Toxicol..

[12] Itoh K., Chiba T., Takahashi S., Ishii T., Igarashi K., Katoh Y., Oyake T., Hayashi N., Satoh K., Hatayama I. (1997). An Nrf2/small Maf heterodimer mediates the induction of phase II detoxifying enzyme genes through antioxidant response elements. Biochem. Biophys. Res. Commun..

[13] Ryter S.W., Alam J., Choi A.M. (2006). Heme oxygenase-1/carbon monoxide: From basic science to therapeutic applications. Physiol. Rev..

[14] Nguyen T., Nioi P., Pickett C.B. (2009). The Nrf2-antioxidant response element signaling pathway and its activation by oxidative stress. J. Biol. Chem..

[15] Kobayashi A., Kang M.-I., Okawa H., Ohtsuji M., Zenke Y., Chiba T., Igarashi K., Yamamoto M. (2004). Oxidative stress sensor Keap1 functions as an adaptor for Cul3-based E3 ligase to regulate proteasomal degradation of Nrf2. Mol. Cell. Biol..

[16] Yang S.-h., Sharrocks A.D., Whitmarsh A.J. (2013). MAP kinase signalling cascades and transcriptional regulation. Gene.

[17] Bloom D.A., Jaiswal A.K. (2003). Phosphorylation of Nrf2 at Ser40 by protein kinase C in response to antioxidants leads to the release of Nrf2 from INrf2, but is not required for Nrf2 stabilization/accumulation in the nucleus and transcriptional activation of antioxidant response element-mediated NAD (P) H: Quinone oxidoreductase-1 gene expression. J. Biol. Chem..

[18] Nguyen T., Sherratt P.J., Huang H.-C., Yang C.S., Pickett C.B. (2003). Increased protein stability as a mechanism that enhances Nrf2-mediated transcriptional activation of the antioxidant response element: Degradation of Nrf2 by the 26 S proteasome. J. Biol. Chem..

[19] Zhang D.D., Hannink M. (2003). Distinct cysteine residues in Keap1 are required for Keap1-dependent ubiquitination of Nrf2 and for stabilization of Nrf2 by chemopreventive agents and oxidative stress. Mol. Cell. Biol..

[20] Huang H.-C., Nguyen T., Pickett C.B. (2002). Phosphorylation of Nrf2 at Ser-40 by protein kinase C regulates antioxidant response element-mediated transcription. J. Biol. Chem..

[21] Joo M.S., Kim W.D., Lee K.Y., Kim J.H., Koo J.H., Kim S.G. (2016). AMPK facilitates nuclear accumulation of Nrf2 by phosphorylating at serine 550. Mol. Cell. Biol..

[22] Qin S., Hou D.X. (2016). Multiple regulations of Keap1/Nrf2 system by dietary phytochemicals. Mol. Nutr. Food Res..

[23] Kumar M., Saurabh V., Tomar M., Hasan M., Changan S., Sasi M., Maheshwari C., Prajapati U., Singh S., Prajapat R.K. (2021). Mango (*Mangifera indica* L.) leaves: Nutritional composition, phytochemical profile, and health-promoting bioactivities. Antioxidants.

[24] Shah K., Patel M., Patel R., Parmar P. (2010). *Mangifera indica* (mango). Pharmacogn. Rev..

[25] Kulkarni V.M., Rathod V.K. (2014). Extraction of mangiferin from *Mangifera indica* leaves using three phase partitioning coupled with ultrasound. Ind. Crops Prod..

[26] Montillet J.L., Cacas J.L., Garnier L., Montané M.H., Douki T., Bessoule J.J., Polkowska-Kowalczyk L., Maciejewska U., Agnel J.P., Vial A. (2004). The upstream oxylipin profile of *Arabidopsis thaliana*: A tool to scan for oxidative stresses. Plant J..

[27] Waridel P., Wolfender J.-L., Lachavanne J.-B., Hostettmann K. (2004). ent-Labdane glycosides from the aquatic plant *Potamogeton lucens* and analytical evaluation of the lipophilic extract constituents of various *Potamogeton* species. Phytochemistry.

[28] McLean S., Reynolds W.F., Tinto W.F., Chan W.R., Shepherd V. (1996). Complete 13C and 1H spectral assignments of prenylated flavonoids and a hydroxy fatty acid from the leaves of Caribbean Artocarpus communis. Magn. Reson. Chem..

[29] Lee S.H., Williams M.V., DuBois R.N., Blair I.A. (2003). Targeted lipidomics using electron capture atmospheric pressure chemical ionization mass spectrometry. Rapid Commun. Mass Spectrom..

[30] Ko Y.-C., Choi H.S., Kim J.-H., Kim S.-L., Yun B.-S., Lee D.-S. (2020). Coriolic Acid (13-(S)-Hydroxy-9 Z, 11 E-octadecadienoic Acid) from Glasswort (*Salicornia herbacea* L.) Suppresses Breast Cancer Stem Cell through the Regulation of c-Myc. Molecules.

[31] Yuki K., Ikeda M., Miyamoto K., Ohno O., Yamada K., Uemura D. (2012). Isolation of 9-hydroxy-10 E, 12 Z-octadecadienoic acid, an inhibitor of fat accumulation from Valeriana fauriei. Biosci. Biotechnol. Biochem..

[32] Dufour C., Loonis M. (2005). Regio-and stereoselective oxidation of linoleic acid bound to serum albumin: Identification by ESI–mass spectrometry and NMR of the oxidation products. Chem. Phys. Lipids.

[33] Kim Y.I., Hirai S., Takahashi H., Goto T., Ohyane C., Tsugane T., Konishi C., Fujii T., Inai S., Iijima Y. (2011). 9-oxo-10 (E), 12 (E)-octadecadienoic acid derived from tomato is a potent PPAR α agonist to decrease triglyceride accumulation in mouse primary hepatocytes. Mol. Nutr. Food Res..

[34] Takahashi H., Kamakari K., Goto T., Hara H., Mohri S., Suzuki H., Shibata D., Nakata R., Inoue H., Takahashi N. (2015). 9-Oxo-10 (E), 12 (Z), 15 (Z)-octadecatrienoic acid activates peroxisome proliferator-activated receptor α in hepatocytes. Lipids.

[35] Kim J.-H., Chen C., Kong A.-N.T. (2011). Resveratrol inhibits genistein-induced multi-drug resistance protein 2 (MRP2) expression in HepG2 cells. Arch. Biochem. Biophys..

[36] Murakami N., Shirahashi H., Nagatsu A., Sakakibara J. (1992). Two unsaturated 9*R*-hydroxy fatty acids from the cyanobacterium*Anabaena flos-aquae f. flos-aquae*. Lipids.

[37] Kobayashi Y., Okamoto S., Shimazaki T., Ochiai Y., Sato F. (1987). Synthesis and physiological activities of both enantiomers of coriolic acid and their geometric isomers. Tetrahedron Lett..

[38] Wahyudi L.D., Jeong J., Yang H., Kim J.-H. (2018). Amentoflavone-induced oxidative stress activates NF-E2-related factor 2 via the p38 MAP kinase-AKT pathway in human keratinocytes. Int. J. Biochem. Cell Biol..

[39] Jeong J., Wahyudi L.D., Keum Y.-S., Yang H., Kim J.-H. (2018). E-p-Methoxycinnamoyl-α-l-rhamnopyranosyl Ester, a Phenylpropanoid Isolated from *Scrophularia buergeriana*, Increases Nuclear Factor Erythroid-Derived 2-Related Factor 2 Stability by Inhibiting Ubiquitination in Human Keratinocytes. Molecules.

[40] Wang R., Kern J.T., Goodfriend T.L., Ball D.L., Luesch H. (2009). Activation of the antioxidant response element by specific oxidized metabolites of linoleic acid. Prostaglandins Leukot. Essent. Fat. Acids.

[41] Akazawa-Ogawa Y., Shichiri M., Nishio K., Yoshida Y., Niki E., Hagihara Y. (2015). Singlet-oxygen-derived products from linoleate activate Nrf2 signaling in skin cells. Free Radic. Biol. Med..

