# Supplementary material for: Activation of Nuclear Factor Erythroid 2-Related Factor-2 by Oxylipin from Mangifera indica Leaves
Source: Antioxidants (Basel). 2024 Sep 16;13(9):1119. doi: 10.3390/antiox13091119 (PMC11428645; doi:10.3390/antiox13091119)
Supplement: Supplementary file 1 [file antioxidants-13-01119-s001.zip › antioxidants-3182581-supplementary.pdf]

Supplementary data

Activation of Nuclear Factor Erythroid 2-related Factor-2 by Oxylipin from *Mangifera indica* leaves

Supplementary data contains 14 figures;

Figure S1. Negative ion mass spectrum acquired by Q-TOF/MS and Q-TOF/MSMS (HR-ESI) analysis of compound **1**.

Figure S2. The ^{1}H NMR spectrum of compound **1**

Figure S3. Negative ion mass spectrum acquired by Q-TOF/MS and Q-TOF/MSMS (HR-ESI) analysis of compound **2**.

Figure S4. The ^{1}H NMR spectrum of compound **2**

Figure S5. Negative ion mass spectrum acquired by Q-TOF/MS and Q-TOF/MSMS (HR-ESI) analysis of compound **3**.

Figure S6. The ^{1}H NMR spectrum of compound **3**

Figure S7. Negative ion mass spectrum acquired by Q-TOF/MS and Q-TOF/MSMS (HR-ESI) analysis of compound **4**.

Figure S8. The ^{1}H NMR spectrum of compound **4**

Figure S9. Negative ion mass spectrum acquired by Q-TOF/MS and Q-TOF/MSMS (HR-ESI) analysis of compound **5**.

Figure S10. The ^{1}H NMR spectrum of compound **5**

Figure S11. Negative ion mass spectrum acquired by Q-TOF/MS and Q-TOF/MSMS (HR-ESI) analysis of compound **6**.

Figure S12. The ^{1}H NMR spectrum of compound **6**

Figure S13. Negative ion mass spectrum acquired by Q-TOF/MS and Q-TOF/MSMS (HR-ESI) analysis of compound **7**.

Figure S14. Negative ion mass spectrum acquired by Q-TOF/MS and Q-TOF/MSMS (HR-ESI) analysis of compound **8**.

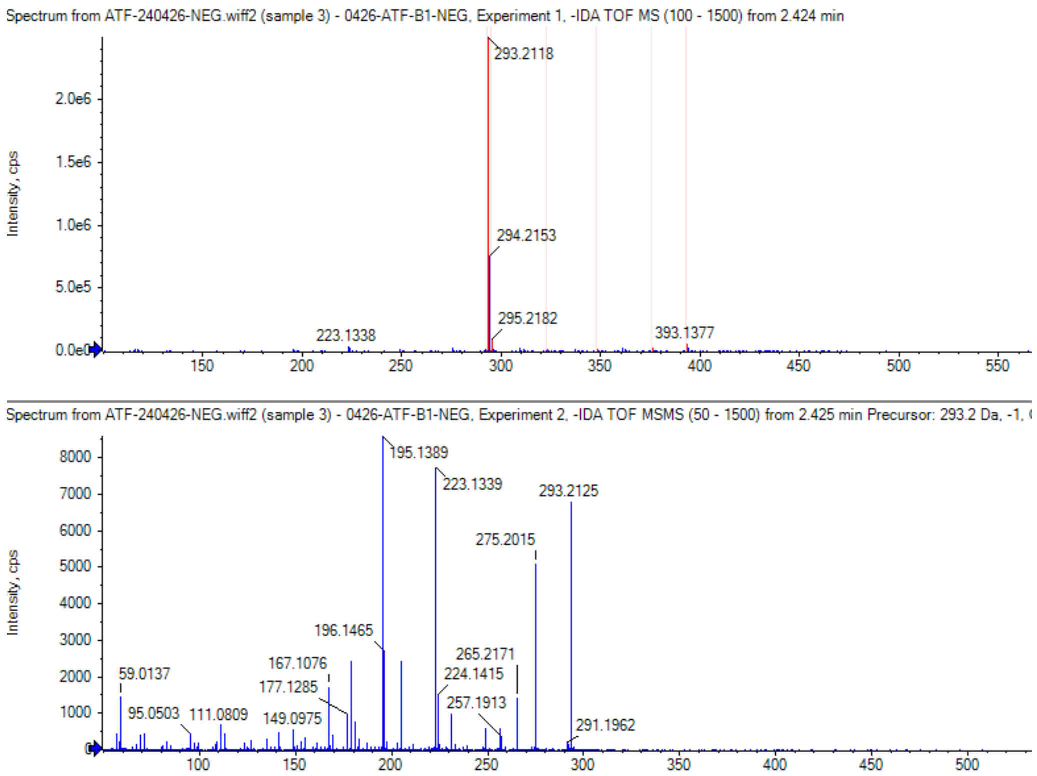

Figure S1. Negative ion mass spectrum acquired by Q-TOF/MS and Q-TOF/MSMS (HR-ESI) analysis of compound **C-1**.

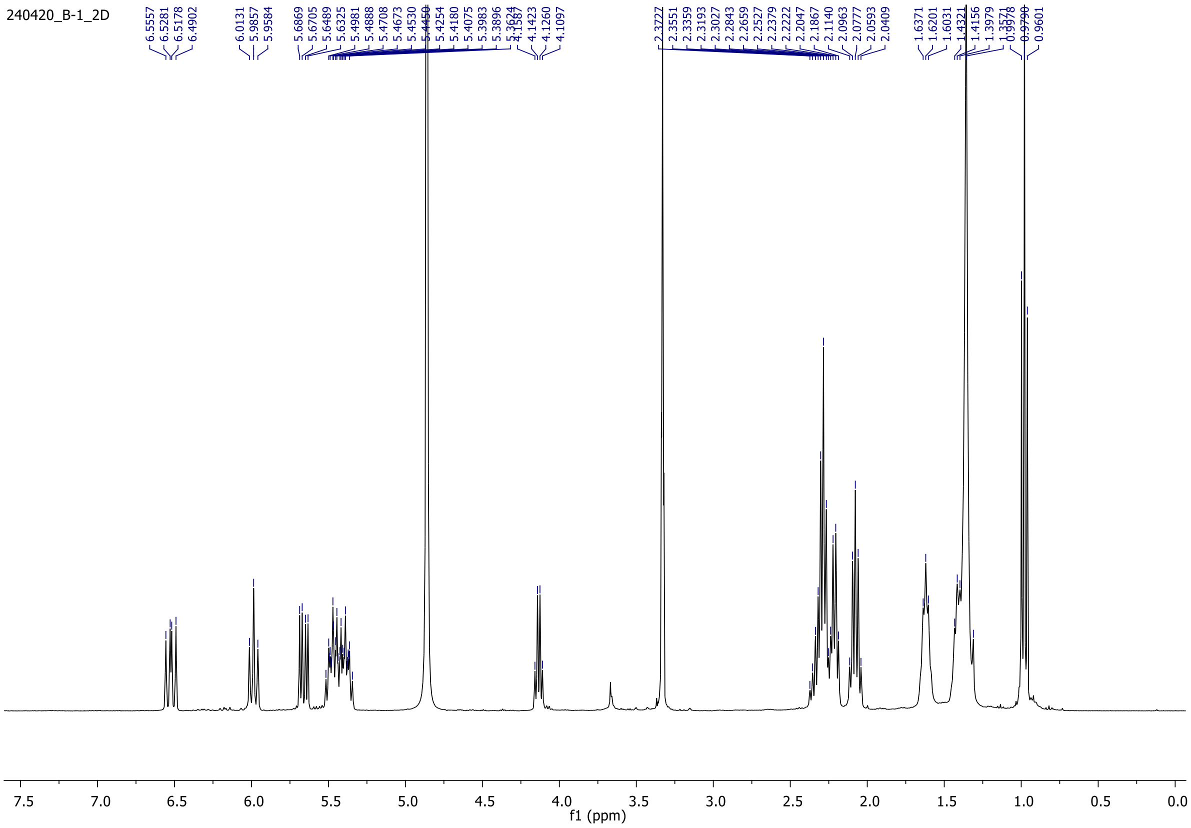

Figure S2. ^{1}H-NMR spectrum of compound **C-1**. (400 MHz, MeOD)

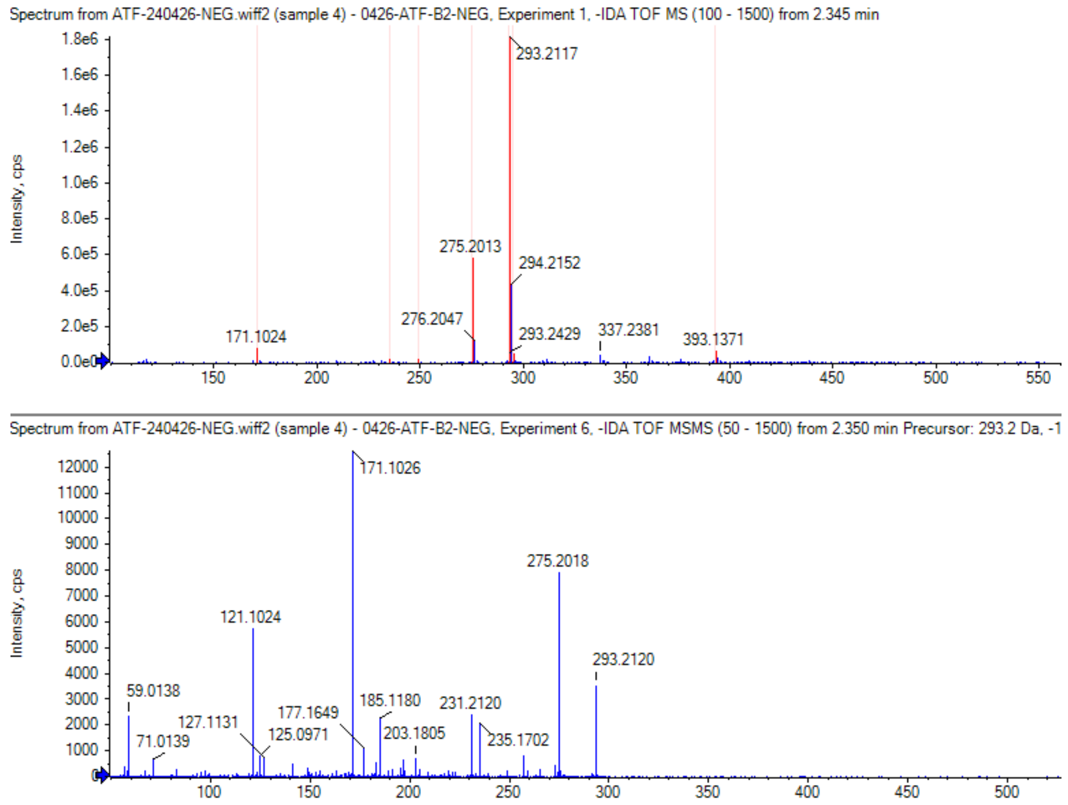

Figure S3. Negative ion mass spectrum acquired by Q-TOF/MS and Q-TOF/MSMS (HR-ESI) analysis of compound **C-2**.

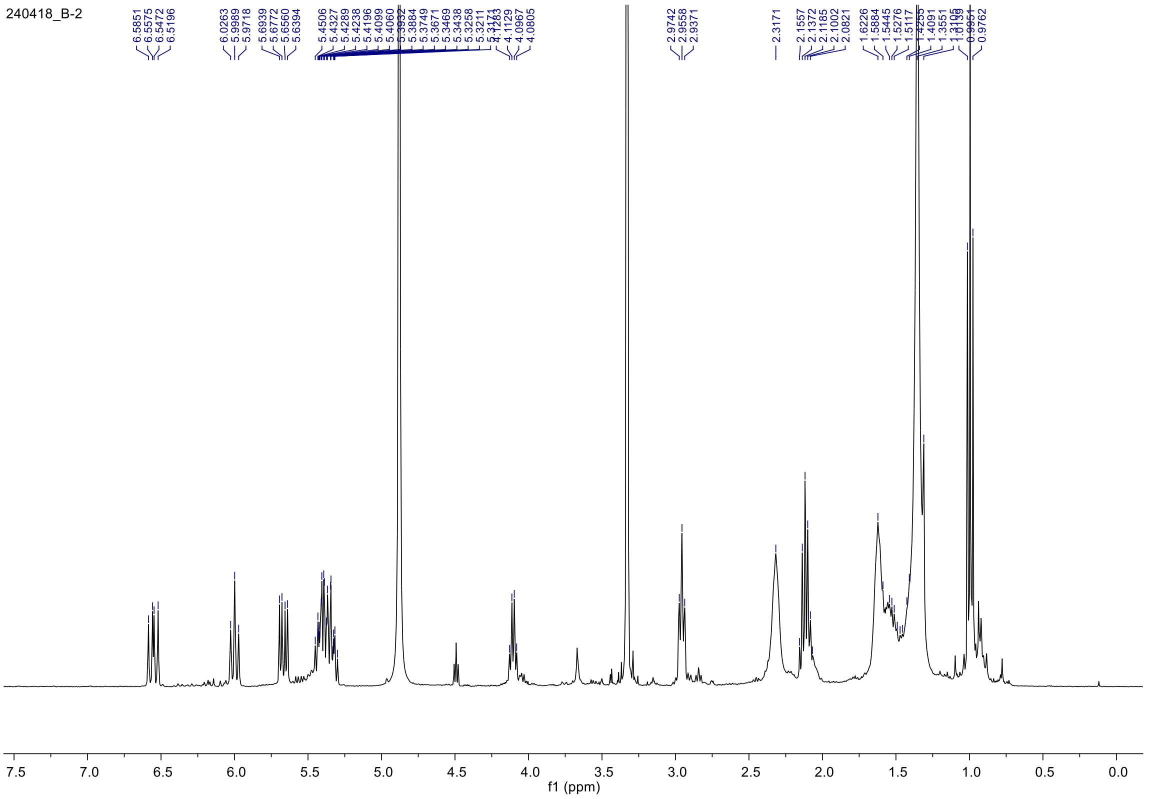

Figure S4. ^{1}H-NMR spectrum of compound **C-2**. (400 MHz, MeOD)

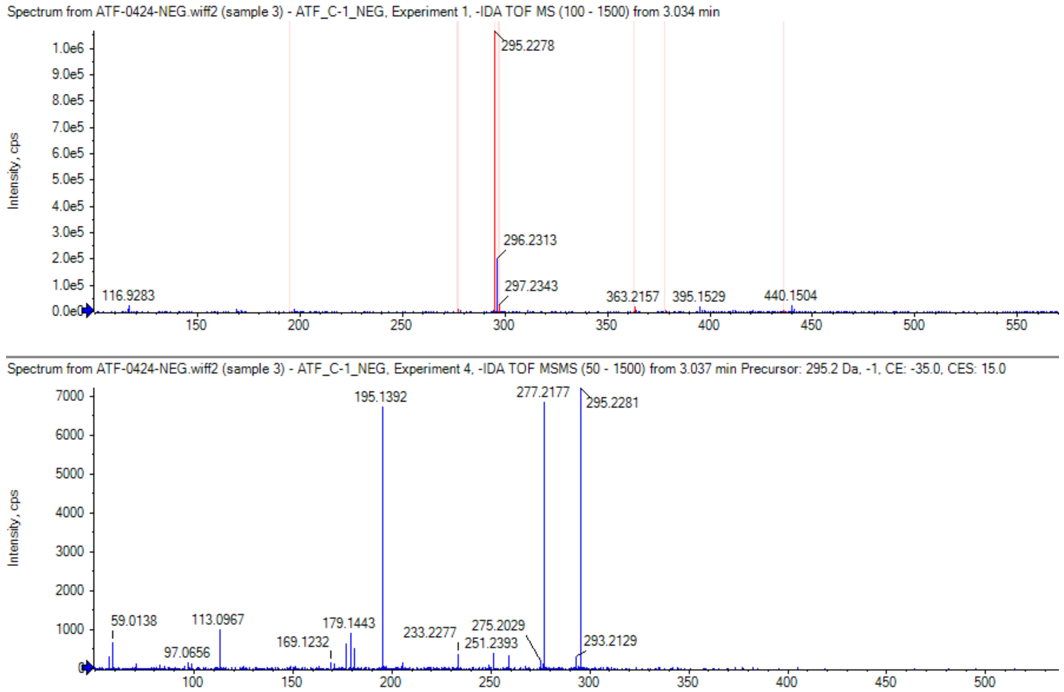

Figure S5. Negative ion mass spectrum acquired by Q-TOF/MS and Q-TOF/MSMS (HR-ESI) analysis of compound **C-3**.

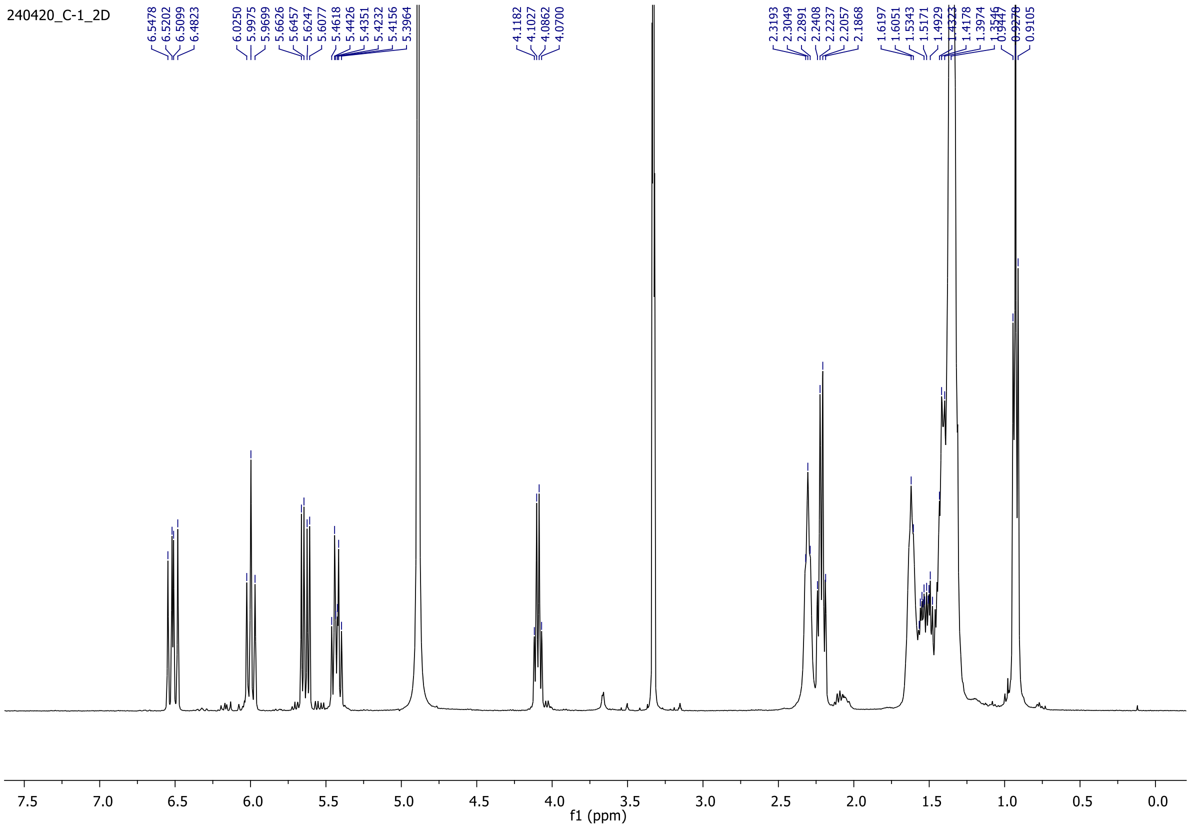

Figure S6. ^{1}H-NMR spectrum of compound **C-3**. (400 MHz, MeOD)

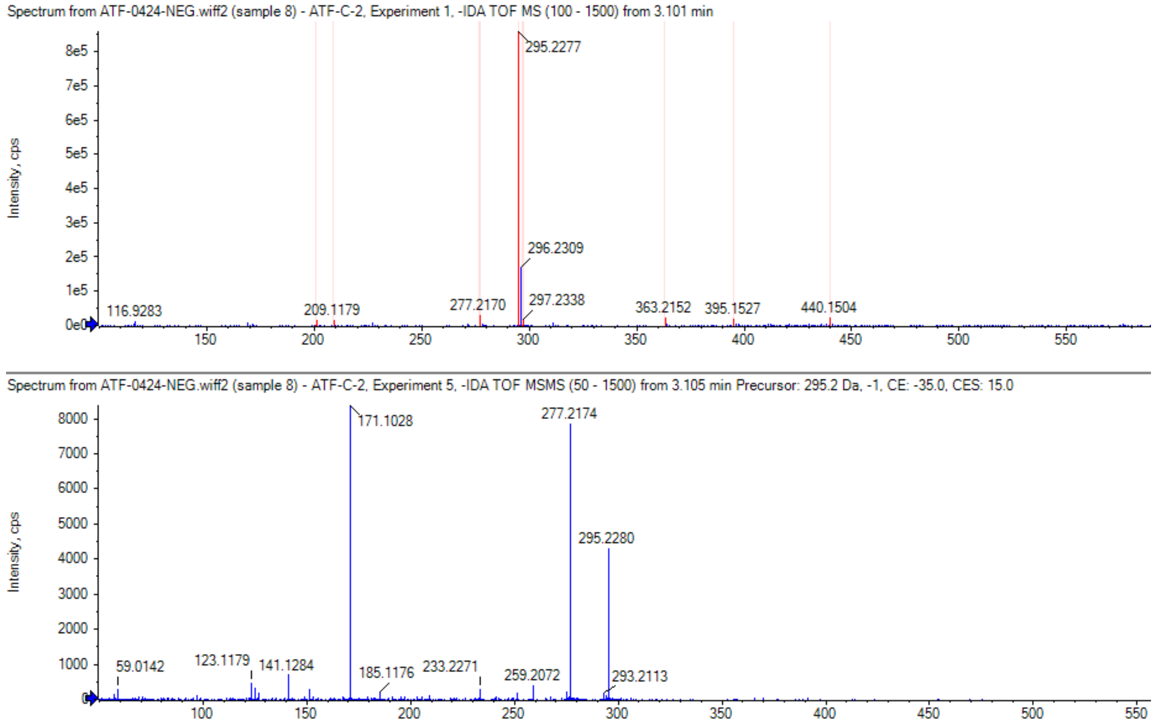

Figure S7. Negative ion mass spectrum acquired by Q-TOF/MS and Q-TOF/MSMS (HR-ESI) analysis of compound **C-4**.

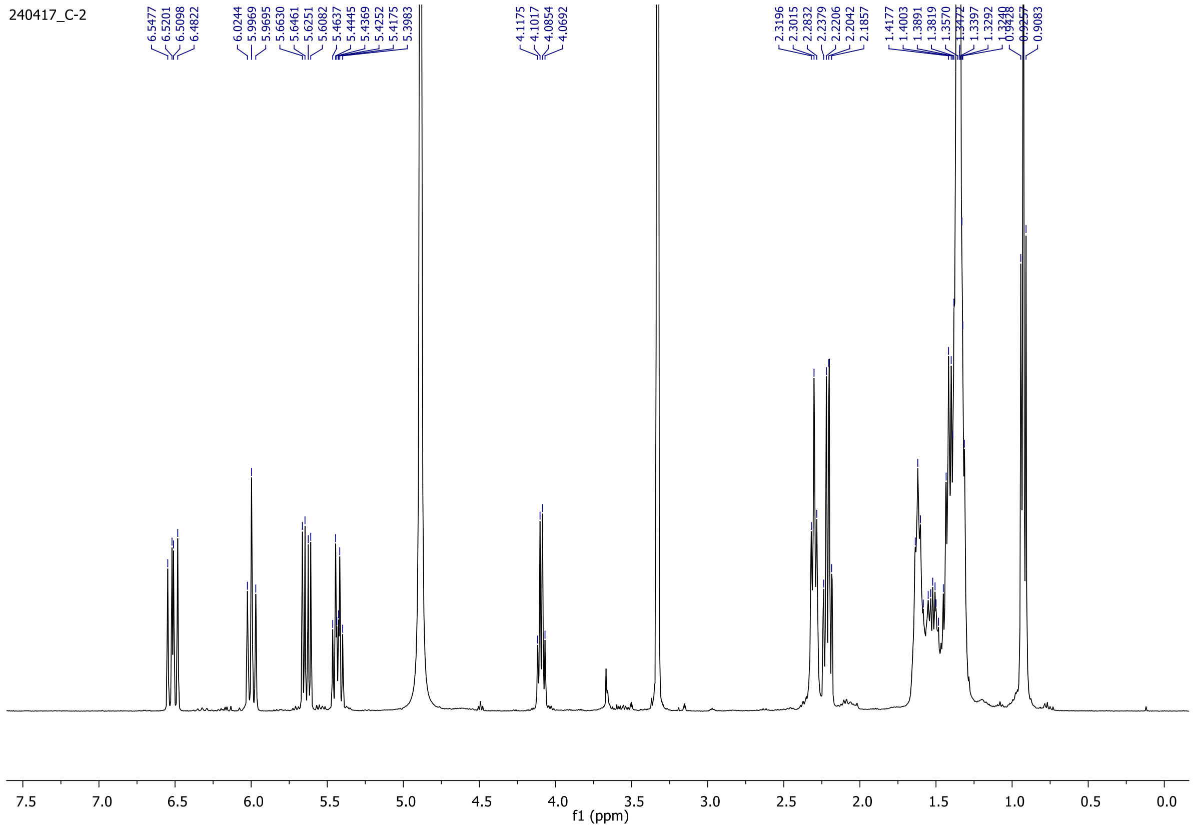

Figure S8. ^{1}H-NMR spectrum of compound **C-4**. (400 MHz, MeOD)

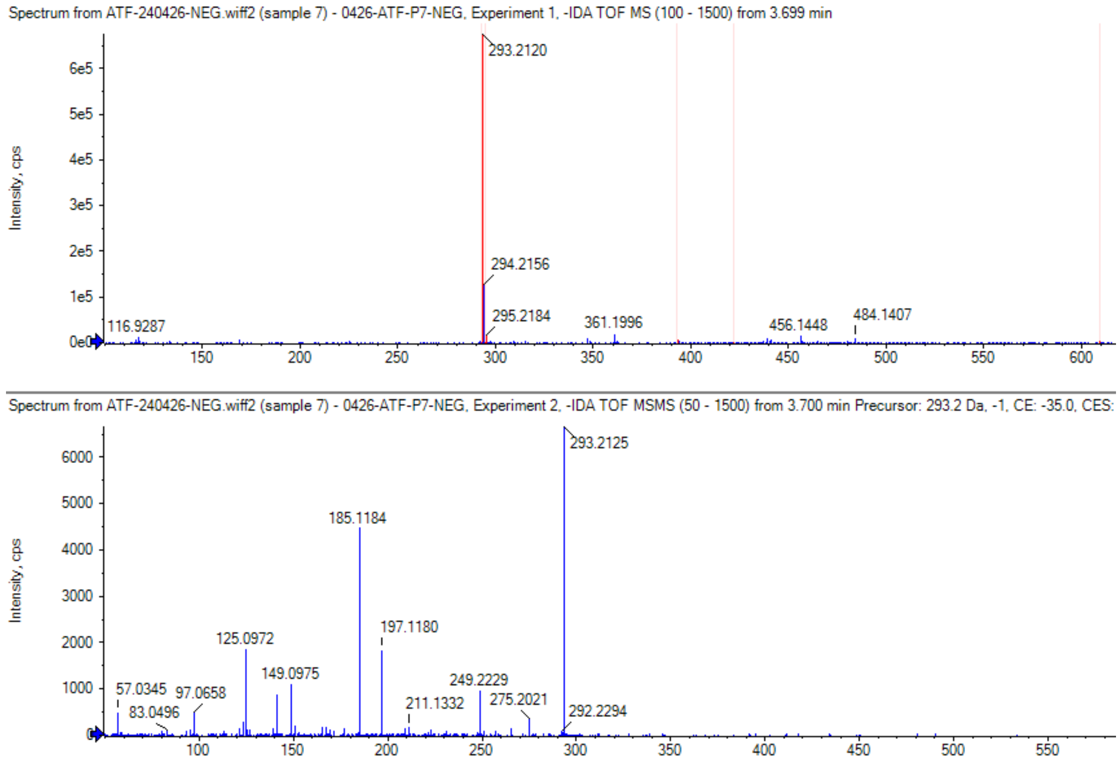

Figure S9. Negative ion mass spectrum acquired by Q-TOF/MS and Q-TOF/MSMS (HR-ESI) analysis of compound **C-5**.

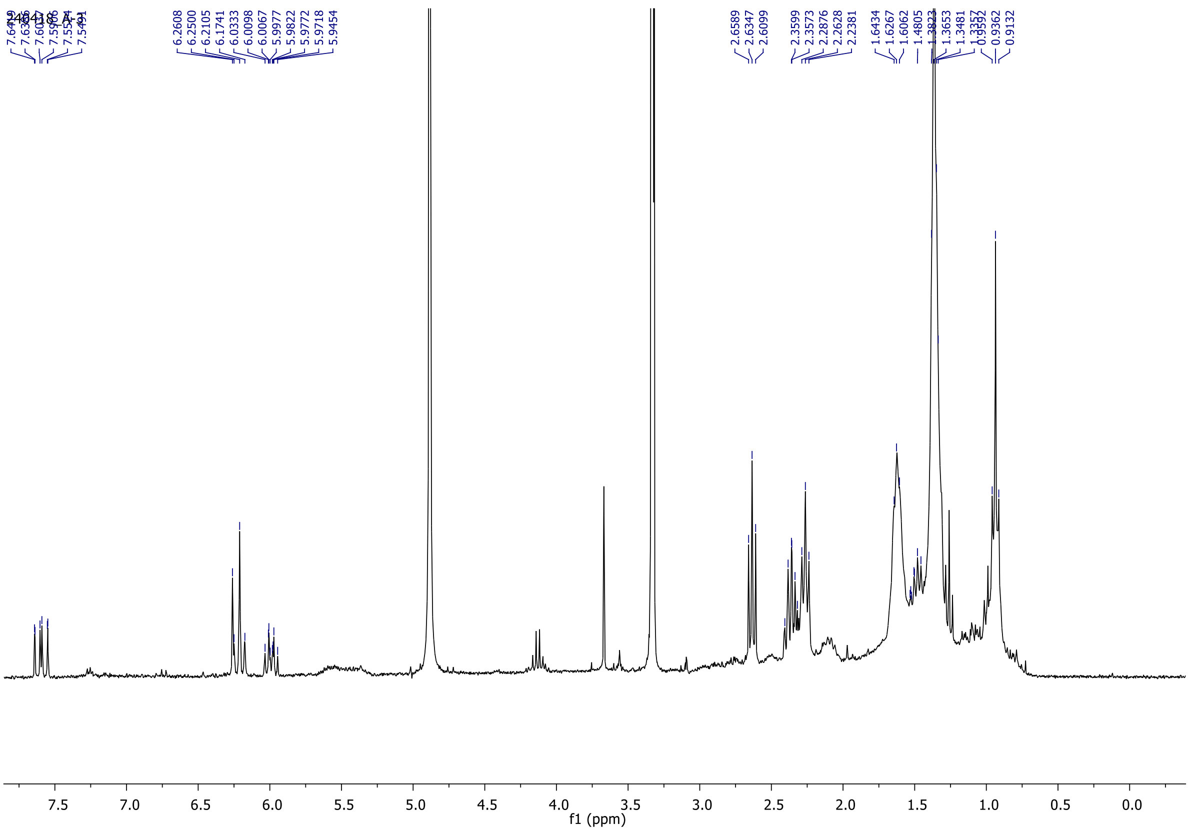

Figure S10. ^{1}H-NMR spectrum of compound **C-5**. (300 MHz, MeOD)

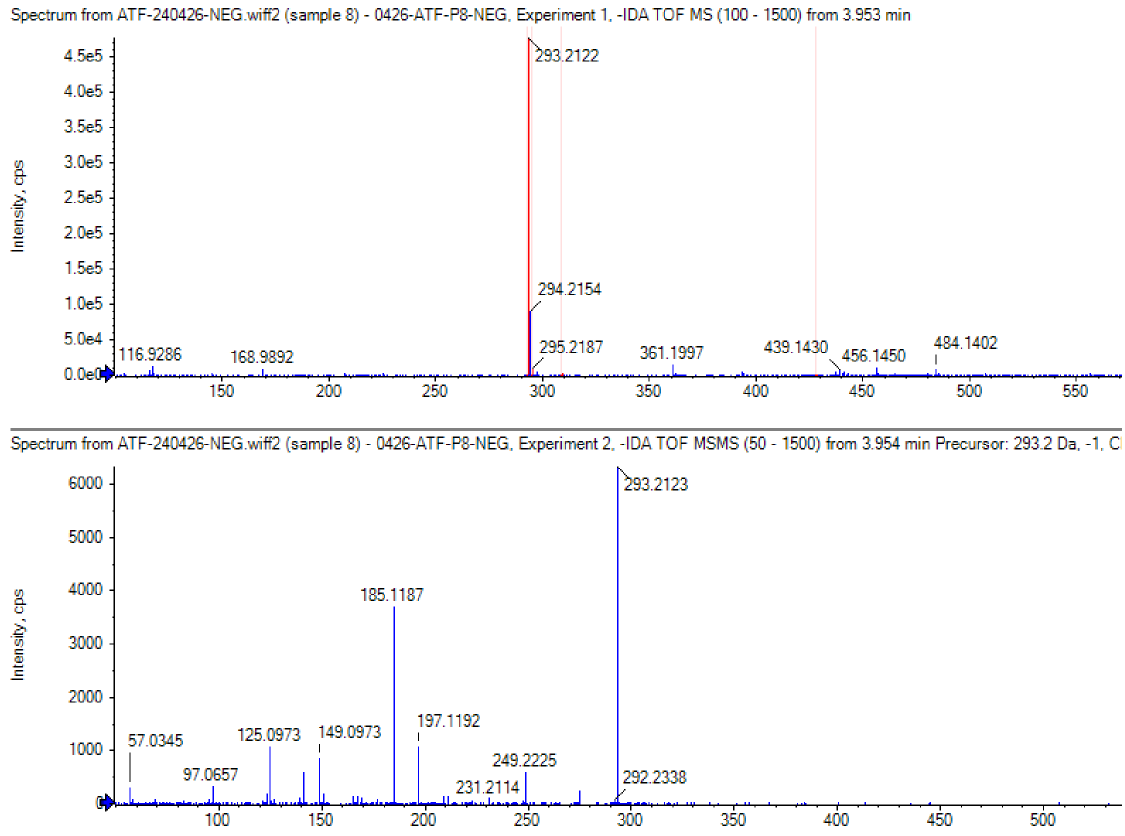

Figure S11. Negative ion mass spectrum acquired by Q-TOF/MS and Q-TOF/MSMS (HR-ESI) analysis of compound **C-6**.

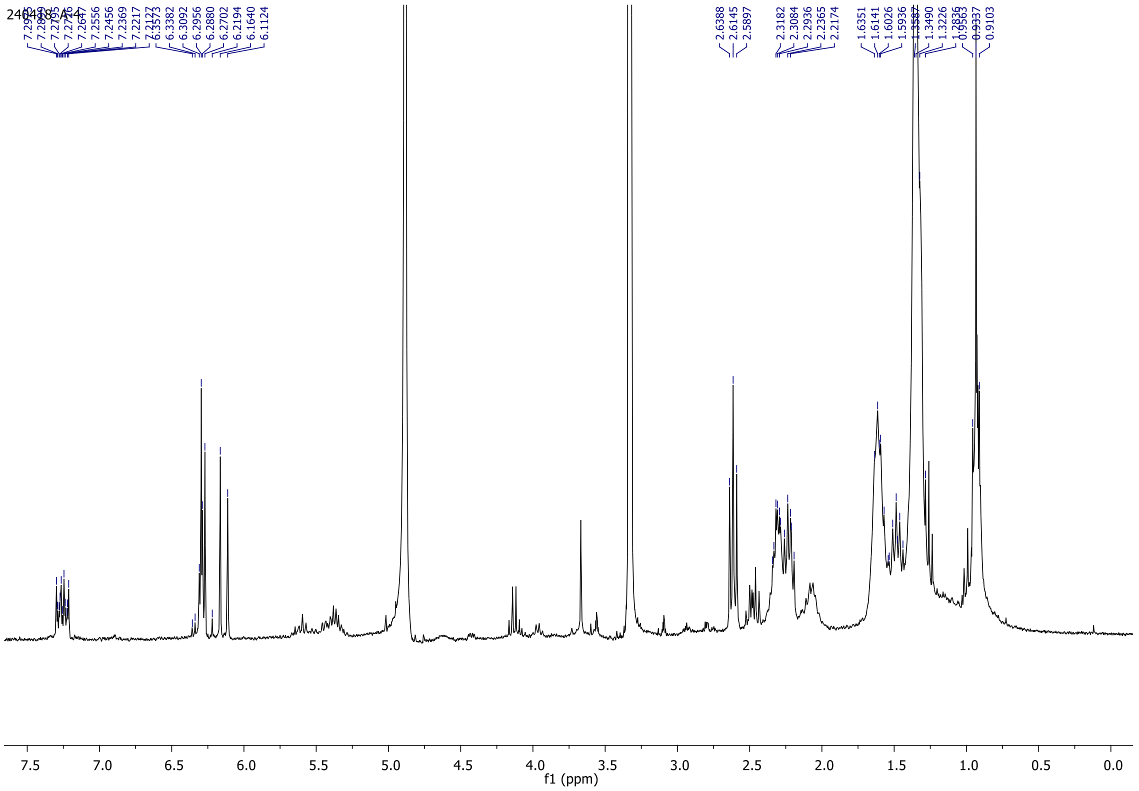

Figure S12. ^{1}H-NMR spectrum of compound **C-6**. (300 MHz, MeOD)

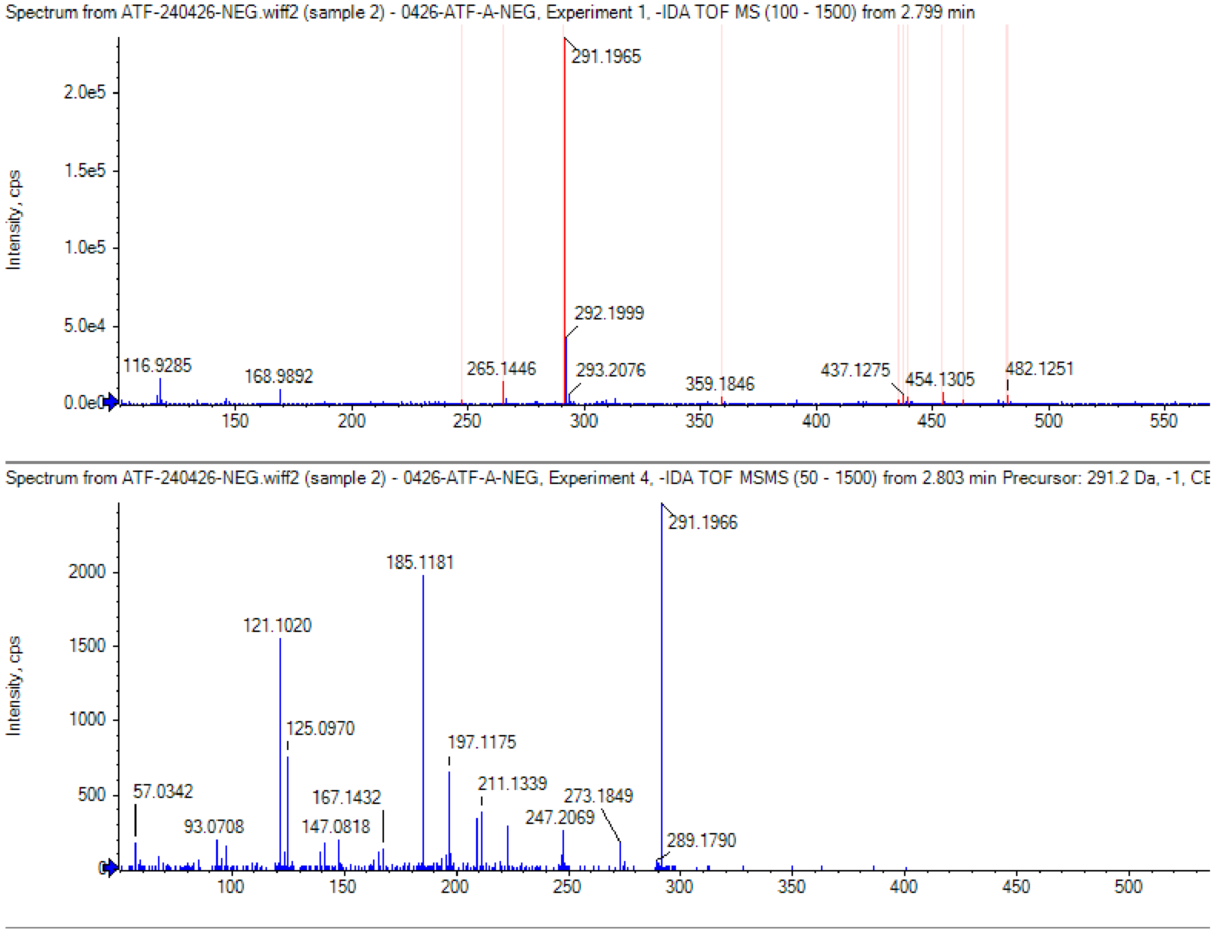

Figure S13. Negative ion mass spectrum acquired by Q-TOF/MS and Q-TOF/MSMS (HR-ESI) analysis of compound **C-7**.

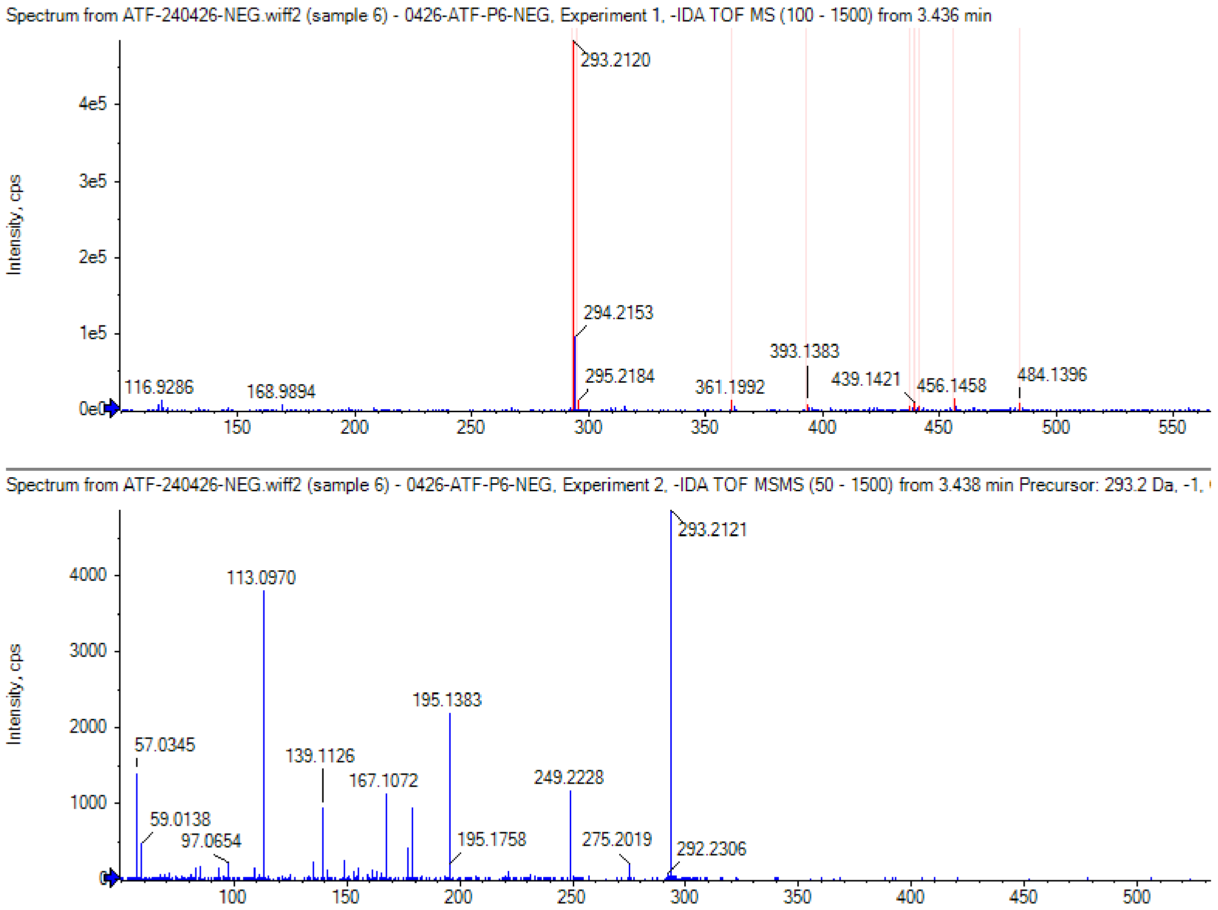

Figure S14. Negative ion mass spectrum acquired by Q-TOF/MS and Q-TOF/MSMS (HR-ESI) analysis of compound **C-8**.
